# The social and environmental impacts on local residents from tourist accommodation near protected areas

**DOI:** 10.1007/s00267-026-02385-5

**Published:** 2026-01-30

**Authors:** Yuqi Zhang, Philippe Hanna, Frank Vanclay

**Affiliations:** 1 Work conducted while at the Department of Cultural Geography, Faculty of Spatial Sciences, University of Groningen, Groningen, The Netherlands; 2 Department of Spatial Planning & Environment, Faculty of Spatial Sciences, University of Groningen, Groningen, The Netherlands; 3 Urban & Regional Studies Institute, Faculty of Spatial Sciences, University of Groningen, Groningen, The Netherlands; 4 Present Address: the College of Landscape Architecture at the Nanjing Forestry University, Nanjing, China

**Keywords:** Social impact assessment, Social impacts of tourism, Protected area management, Tourism management, Tourist-host interaction, conservation induced displacement and resettlement, Ecotourism

## Abstract

Tourism is often promoted as a strategy for offsetting the social impacts on the lives and livelihoods of people in nearby host communities from conservation actions and the establishment of protected areas and national parks. However, the presence of tourists also creates significant negative social and environmental impacts on local people and communities. We examine the impacts created by rural tourism operators (specifically boutique rural hotels and bed-and-breakfast enterprises) on the residents of Longweiba village in the Wulingyuan Historic and Scenic Area, Hunan Province, China. The management arrangements for this World Heritage Site mean that residents are regulated by different management regimes depending on whether they are inside or outside the high conservation zone, which has created inequity and conflict. Using an ethnographic approach and interviews with 30 local residents and one government official, our results reveal that, while tourism has contributed to local economic development and improved the living environment, it has also led to a range of concerns about: protected area management; various environmental issues; inequitable regulations; inconsistent governance policies; unfair building restrictions; sewage; odor pollution; water shortages; and a fear of involuntary resettlement. The poor planning of tourism, inequitable policies, and inconsistent enforcement of regulations have exacerbated these issues. We conclude that, while tourism has some benefits for host communities, better governance and environmental management, more inclusive community engagement, and clear consistent policies are needed to ensure that tourism development within or near protected areas is sustainable and equitable.

## Introduction

Although somewhat contested, the increased tourism brought about by the designation of protected areas generally benefits host communities, including by: community empowerment, positive economic development, reduced poverty, and enhanced community wellbeing (Mason and Cheyne 2000; Sharpley 2002; Telfer and Sharpey 2008; Kim and Jamal 2015; Lane and Kastenholz 2015). In many countries, tourism is considered to be a sustainable development strategy for rural areas (Su 2011; Vanclay 2011; Kaptan Ayhan et al. 2020; Ruiz-Real et al. 2022). However, as discussed in this paper and elsewhere, tourism to protected areas and rural tourism generally can also have many negative social and environmental impacts (Vanclay 2017; Pope et al. 2019; Guo and Jordan 2022; Stoffelen and Ioannides 2022; Kahangirwe et al. 2025).

Over the last three decades or so, China has had a rapid increase in the number of protected areas (Zhang et al. 2025a), and an increasing amount of rural tourism. While rural hotels provide accommodation, they generally also promote the special characteristics of the local environment, especially local food and culture and natural landscapes (Chen et al. 2013; Santana-Jiménez et al. 2015). Rural hotels usually provide opportunities for visitors to experience rural life and interact closely with local people (Cai et al. 2021). However, such immersive experiences can have negative social impacts on host communities (Wall and Mathieson 2006; McCombes et al. 2015; Zhang et al. 2019; McCombes and Vanclay 2022; Liu et al. 2023; Tian et al. 2023,2024).

With the growing interest in rural tourism, the Chinese Government has been advocating for more entrepreneurialism, and a concept of ‘enterprise+farmer’ has been advocated (meaning a partnership between farmers and entrepreneurs). This has led to a dramatic increase in the number and types of rural accommodation providers (Huang 2020; Qiao et al. 2021; Liu et al. 2023), including Bed & Breakfast (B&B) operations as well as boutique rural hotels that are often financed by external investors, mostly in cooperation with local people and local government.

Despite rural tourism potentially contributing to poverty alleviation (Medina-Muñoz et al. 2016), boutique rural hotels can also trigger local social conflict, and can undermine community cohesion because they make the inequality between wealthy tourists and poor people in host communities more obvious, potentially causing resentment and jealousy (Wall and Mathieson 2006; Kontogeorgopoulos et al. 2015; Liu et al. 2023). The primary purpose of this paper, therefore, is to better understand how tourist accommodation providers in or near protected areas create impacts on host communities and local people. From our research in the Wulingyuan World Heritage Site in China, we extrapolate to the broader discussions about the management of the environmental and social impacts of tourism in protected areas, and of rural tourism generally.

We use Longweiba village in the Wulingyuan World Heritage Site as our case study. Wulingyuan is a very scenic location and is gaining much attention in China and internationally (Zhu et al. 2024). Longweiba is a linear village that straddles one of the gateways to a core protection zone of Wulingyuan (Zhang et al. 2025b). Longweiba has recently experienced rapid growth in tourism, with many local people establishing a B&B or boutique rural hotel. A particular characteristic of Longweiba is that the village is split between those who live inside the gate (high protection zone) and are severely constrained in what they can do, and those who live outside the gate and have relative freedom, including in their ability to provide tourist accommodation facilities.

We used qualitative methods to identify the social impacts that host communities experience from the rapid development of rural tourist accommodation. We applied a ‘social impact assessment lens’ (Vanclay 2002, 2012, 2020) to our research. Although the discourse of the ‘social impacts of tourism’ (Deery et al. 2012; Sharpley 2014) suggests many of the social issues arising from tourism, the discourse of ‘social impact assessment’ potentially provides a practical approach to managing these social impacts (McCombes et al. 2015; McCombes and Vanclay 2022). By identifying the full range of host community issues and perceptions, the social impact assessment discourse provides a conceptual understanding of the social impacts of rural tourism on local communities.

## The social impacts of tourism to protected areas

The social impacts of tourism have been much discussed (Deery et al. 2012; Almeida García et al. 2015; McCombes et al. 2015; Scholtz and Slabbert 2018; Kahangirwe et al. 2025). ‘Social impacts’ have been defined as all the social, cultural and socio-psychological consequences experienced by people, at all levels (e.g. individual, family/household, social group, community/society), and in corporeal (physical), cognitive (perceptual) or affective (emotional) ways (Vanclay 2002, 2024a). The experience of social impacts affects the ways people live, work, play, relate to one another on a day-to-day basis, organize to meet their needs, and generally cope as members of society (Burdge and Vanclay 1996; Vanclay 2002, 2020; Edelstein and Vanclay 2024).

Tourism to protected areas is inevitably interconnected with the economic, social, cultural, and environmental dimensions of local communities (Aswani et al. 2015; Yang et al. 2021; Dai et al. 2023). A close connection between local people and their environment is typical of rural communities everywhere (Vanclay 2008; Carneiro et al. 2015). The perceived potential economic benefits from tourism encourages many local people to become involved in providing tourism services. Other positive social impacts from tourism include: local economic development; increased employment opportunities; empowerment of host communities and women; and improved infrastructure (Andriotis 2005; Su et al. 2019; Liasidou et al. 2021; Stoffelen and Ioannides 2022; Su et al. 2023). However, tourism to protected areas and rural tourism generally also have negative impacts on host communities, including: in-migration; increased cost of living; an increasing perception of relative deprivation; damage to the local environment; increased demand on local services; overcrowding and overtourism; unfair distribution of costs and benefits; a clash of values and culture; sex work; increased substance use; changes in livelihood activities; the seasonality of tourism work and poor working conditions; rapid community change and loss of sense of place; and nuisance and annoyance (Vanclay 2002; Jago et al. 2006; Frauman and Banks 2011; McCombes et al. 2015; Li et al. 2020; Diaz-Parra and Jover 2021; Wang 2021; McCombes and Vanclay 2022; Kahangirwe et al. 2025). These positive and negative social impacts are context specific, and unequally affect members of a given community (Vanclay 2002, 2012; Stoffelen and Ioannides 2022). The impacts are influenced by the particular characteristics of the destination and its social setting (Tosun 2002). At various times over the last 50 years or so, growing concern about the impacts of tourism has led to the development of concepts such as ‘overtourism’, ‘tourism carrying capacity’, ‘social carrying capacity’, and ‘multiple carrying capacities’ (McCool and Lime 2001; Zhong et al. 2011; Salerno et al. 2013; Milano et al. 2019).

How to effectively manage the social impacts of rural tourist accommodation needs greater consideration, particularly because the intensity and intimacy (closeness) of the tourist-host encounter (immersiveness) has much potential to create harm as well as good (McCombes et al. 2015; McCombes and Vanclay 2022). However, most research into rural accommodation has primarily focused on their operational strategies, tourist behavior, and tourist experiences, with insufficient attention being given to the social and environmental impacts of tourism on host communities.

Although some research has suggested tourist accommodation provides positive economic impacts on host communities (Pasanchay and Schott 2021; Liu et al. 2023) and offers opportunities for promoting local culture and lifestyles (Wang 2007), others argue that rural tourism has worsened social and economic inequalities (Kontogeorgopoulos et al. 2015). In China, rural tourist accommodation operations have impaired social relations in some communities, leading to distrust and a sense of exclusion among some people (Li et al. 2023, 2024; Liu et al. 2023). While the social impacts of rural tourism have been discussed in the tourism literature to some extent, various related topics have not been adequately addressed in practice (although some references can be found), including: embedding rural accommodation in the local social setting (Liu et al. 2023); the role of rural tourism in poverty alleviation and in promoting sustainable livelihoods (Pasanchay and Schott 2021); and the practical challenges of establishing B&Bs and other forms of rural tourism (Kunjuraman and Hussin 2017).

## Background information about Wulingyuan and Longweiba village

**Fig. 1:**
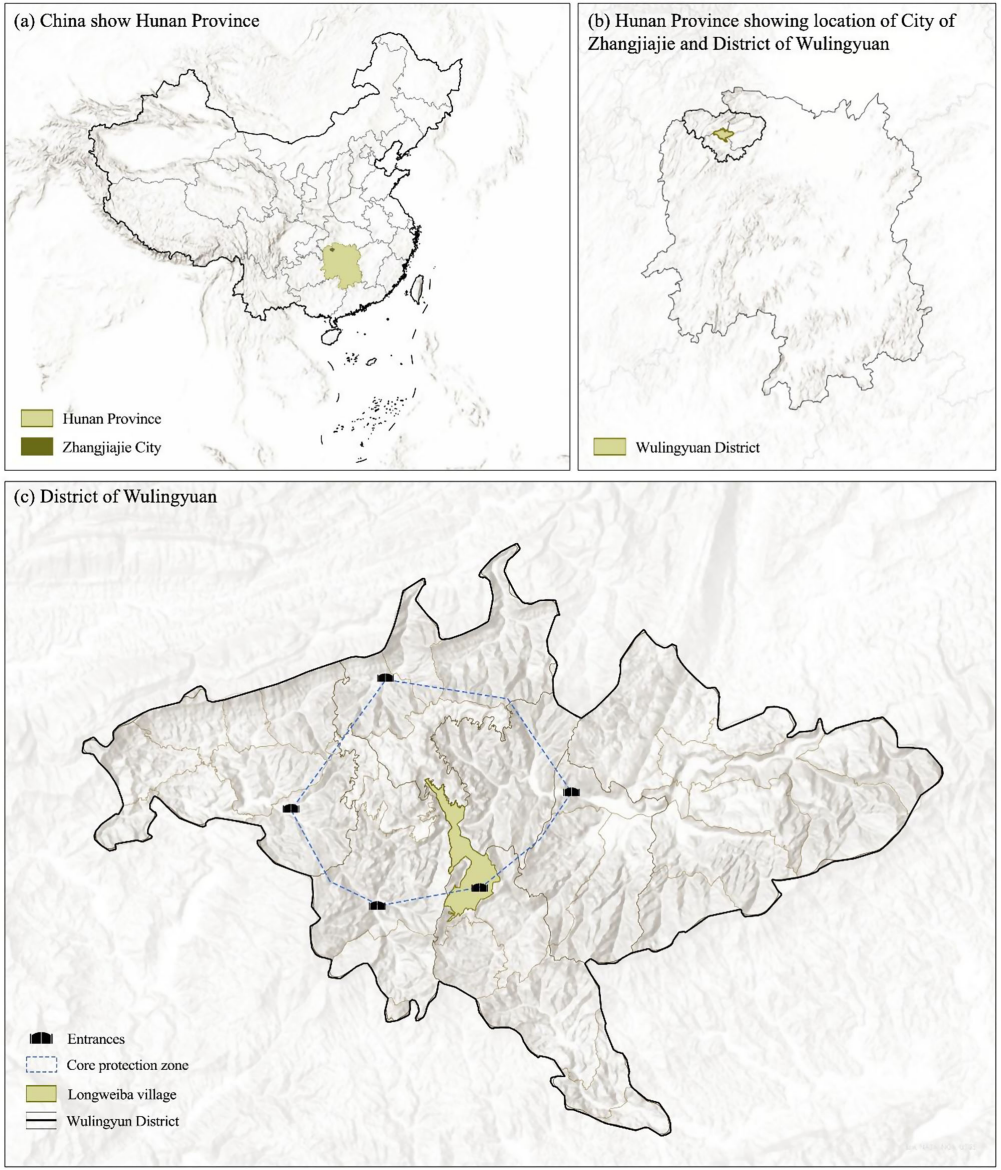
The location of Wulingyuan and the spatial relationship between Longweiba and the entry gates to high protection zones within the World Heritage Site. **a** Location of Hunan province in China; **b** location of Zhangjiajie city and Wulingyuan district in Hunan province; and **c** district of Wulingyuan. Source: basemap data © Esri, USGS, accessed via ArcGIS.

The Wulingyuan World Heritage Site is also a UNESCO Global Geopark, and in China it is known as the ‘Wulingyuan Historic and Scenic Area’. For convenience, in referring to the general area, we shall simply use ‘Wulingyuan’ in this paper. Wulingyuan is located in eastern China (see Fig. 1). It is famous for its unique quartzite sandstone landscape. It received 4.29 million visitors during 2019 (Statistics Bureau of Wulingyuan District 2020). The region is ecologically significant, having over 3000 plant species and 195 vertebrate species. There are 9 rare and 3 vulnerable plant species and 30 protected animal species (IUCN 1992; MHURD 2005; Chen 2020). With a total area of 397 square kilometers, the site encompasses 33 administrative districts and has a total population of approximately 55,000 people, 94% percent of whom are from the Tujia ethnic minority group.

Longweiba is one of China’s 1399 national key rural tourism villages, and is a traditional ethnic Tujia village, with a registered population of 648 people (MCT 2022). Longweiba is a dispersed linear village of about 5 hamlets spread along an escarpment. Actually, Longweiba’s Chinese name (**龙尾巴**) means ‘dragon’s tail’ (see Fig. 1). In the middle of Longweiba is the Zimugang Entrance, one of five entry gates to the core protection zones and major tourism areas of Wulingyuan. The Zimugang Entrance effectively splits the hamlets comprising Longweiba between those located inside and outside the gate. This entrance gate has profound implications on local residents (as we discuss in our results section and in Zhang et al. 2025b).

The Zimugang Entrance was constructed in 2002. Before 2002, all residential areas were outside the controlled area (as it was then). Now, after zoning changes, expansion of the controlled area, and construction of barriers and gates, the residential areas inside and outside the controlled area operate under different management regulations and are governed by different institutions (Su et al. 2017). The residents outside the gate are managed by the local government, while the residents inside the gate are managed by the local government as well as by the Wulingyuan Management Agency, leading to very different conditions and restrictions on activities. The local people call this governance model, ‘one village, two policies’.

People in Longweiba have been involved in providing tourism facilities since the 1980s. However, the negative environmental impacts created by tourism facilities has prompted the various governing bodies to initiate several waves of demolition of buildings inside the gate (especially in 1992, 2000, and 2008) (Li et al. 2024). Because of this, for several years there were no new tourism developments in Longweiba. However, with the increasing tourism to Wulingyuan, a local family outside the gate renovated their house and established the first B&B in Longweiba in 2015. This was soon followed by several more B&Bs being established. Due to Wulingyuan’s outstanding scenic beauty and local cultural heritage, Longweiba became designated as a Key National Rural Tourism Village in 2020, which increased its appeal as a tourism destination. By 2023, Longweiba had also become a model village for rural revitalization (Red Net 2023). Longweiba now has several boutique rural hotels, leading to the village gaining a reputation as a ‘high-end B&B community’. The differential management regulations between the residential areas inside and outside the gate, coupled with the rapid development of tourism, have led to significant negative social impacts, and to concerns about a lack of fairness, especially inside the gate. This has affected how people feel about tourism. To improve policymaking, a better understanding of local people’s perspectives about tourism development is needed.

Although Longweiba has been a gateway community to Wulingyuan since construction of the Zimugang Entrance in 2002, until recently its relatively remote location has resulted in limited tourism. In the interests of park management and environmental restoration, the local government has been relocating people and demolishing buildings inside the gate, and has imposed various restrictions on building activity, making the renovation or expansion of houses nearly impossible (Li et al. 2024). Thus, no tourism enterprises were established in Longweiba between 2002 and 2015. However, as mentioned above, in 2015 some residents outside the gate renovated their house and established the first B&B in the village, which gradually encouraged other villagers to also establish B&B operations. By September 2022, when the fieldwork was being undertaken, there were 13 rural accommodation providers in Longweiba and a further 9 buildings being renovated to become rural accommodation. Some were self-operated by local people, while others were being operated in collaboration with outside investors. Most of these accommodation providers were targeting the middle to high-end market, with prices exceeding 1000 CYN (approx. USD $140) per night. Because residents within the controlled area are subject to strict regulations and are not permitted to engage in tourism activities, the development of rural accommodation was limited to those residents living outside the controlled area.

The development of rural accommodation in Longweiba was encouraged by various government policies. In 2013, the central government proposed the ‘Beautiful Countryside’ program, which advocated creating attractive and livable villages by enhancing aesthetic appeal, infrastructure, and public services in rural areas (CPC Central Committee and the State Council 2013). In 2014, the City of Zhangjiajie issued a guidance document, ‘B&B Management Measures’, and gradually relaxed restrictions on housing construction in Longweiba. In 2015, the central government encouraged rural tourism development to alleviate poverty (CPC Central Committee and the State Council 2015), and in 2017, a rural revitalization strategy was introduced to create vibrant, prosperous and sustainable rural communities, and to improve the quality of life of rural residents (CPC Central Committee and the State Council 2018). In 2018, the local government made efforts to develop rural tourism in Longweiba by actively supporting B&Bs (Longweiba Village Committee 2018).

Another explanation for the proliferation of rural accommodation in Longweiba was the fact that some accommodation providers elsewhere in Wulingyuan were being closed down. Since 2015, many tourist facilities in Wulingyuan were closed by the local government for various reasons (including increasing pollution), and people were gradually being resettled outside the controlled area. This closure of facilities created a shortfall in tourist accommodation in the region, and thus there was strong demand for more accommodation, especially with the increasing numbers of tourists. However, the development of rural accommodation in Longweiba faced some challenges. In late 2016, extensive roadworks commenced on the only road leading into Longweiba, which reduced access to the village until completion in 2019. In early 2020, the outbreak of the Covid pandemic caused a complete halt to tourism in China, with significantly reduced tourism continuing up until early 2023, when China lifted all quarantine measures (much later than in many other places in the world) (Chen et al. 2024).

## Methodology

Several qualitative research methods – including field-based observation and semi-structured and indepth interviews – were used to investigate the views of local people in Longweiba about tourism generally, the social impacts they experience from tourism, as well as their expectations and concerns for the future. The interviews primarily focused on the social impacts arising from the tourism accommodation enterprises in the region. They covered several broad topic areas, including: residents’ attitudes towards tourism accommodation; perceived changes in livelihoods; perceived environmental changes associated with tourism accommodation; impacts on social relations and community cohesion; interactions with government and the Wulingyuan Management Agency; and residents’ expectations and concerns regarding future tourism development. The interviews were guided by a flexible interview guide with adaptation to the specific characteristics of each interviewee, particularly whether they were involved in tourism accommodation provision, as well as by their willingness and ability to articulate their experiences and views.

Fieldwork was conducted from July to September 2022. Data was collected face-to-face, with some interviews being semi-structured while others were informal conversations, depending on the interviewee’s experiences and availability. Some interviews took place at the interviewee’s home, while others occurred on the roadside or on farmland. A total of 31 individuals were interviewed, including 19 residents outside the gate (6 of whom were involved in rural accommodation operations), 11 residents inside the gate (none involved in tourism), and one government official. Although only a small number of people were interviewed, in general the characteristics of those who were interviewed were indicative of the then current residents of the village (approx. 300 permanent residents with a registered population of 648 people).

The interviews ranged from 15 to 120 min. Most were audio-recorded, although for two informal interviews only notes were taken. The interviews were conducted in Mandarin Chinese or in a local dialect understandable to the primary author. Recruitment of interviewees was done through convenience sampling and then by snowballing. As is customary in China, the primary author first contacted community officials to arrange interviews with village representatives. She then approached local people in the five hamlets within Longweiba to inquire if they were willing to participate. Willing participants were informed about their rights and advised that the data would be kept anonymously. Informed consent was obtained verbally rather than requiring signatures. The study adhered to the principles of ethical social research (Vanclay et al. 2013) and was approved by the ethics committee of the authors’ institution (the Faculty of Spatial Sciences at the University of Groningen).

Participatory observation and ethnographic methods were also used, with the primary author living in several of the villages in Wulingyuan for a total of six weeks in 2022. For five days, she stayed in Longweiba in a B&B enterprise run by local people, participating in their daily life. Additionally, the primary author observed the lives of local residents, participated in their family gatherings (when invited), and observed their tourism business activities. She took field notes and maintained a research diary. Basic statistical information, such as the number of residents and rural accommodation providers, and economic data about the community were obtained from the village committee. The integrity of the research was established by achieving saturation and by the use of triangulation.

The recorded interviews were transcribed, and the primary author iteratively analyzed the interview data using a combination of open and systematic coding (Hay and Cope 2021) using Atlas.Ti (version 24). The interview transcripts were descriptively coded to identify the recurring themes, including tourism-related concerns (e.g. pollution), benefits (e.g. economic benefits), policies (e.g. relating to house-building, tourism development), and daily activities (e.g. road works, street sweeping). Also, she coded the social impacts and changes people experienced that related to tourism or rural accommodation. In a second round of coding, she refined, re-ordered, or merged the initial codes to create thematic codes based around the social impact categories (e.g. economic, environmental, cultural, political) nominated by Vanclay (2002).

## Results: Resident perspectives on tourism accommodation

### Social impacts arising from tourist accommodation providers

The increase in rural accommodation in Longweiba together with growing tourism to Wulingyuan has brought many changes to the lives and livelihoods of local people. Their traditional subsistence farming way of life has largely been replaced by a livelihood model based on the provision of services to tourists, or by moving to other locations to find work (sometimes a considerable distance away). Together, this has resulted in much abandonment of farmland. Some interviewees indicated that the rural accommodation industry has improved local economic development and has provided residents with local employment and business opportunities. This has prompted some young people who had left the village to return home because of these job opportunities or to establish their own small business ventures. However, as mentioned by some interviewees, ‘boutique rural hotels have both positive and negative aspects’. Local people generally experience disadvantage when seeking employment in the tourism industry due to their lower education levels and lack of hospitality training. Boutique rural hotels tend to hire skilled and experienced individuals from outside the region, leaving local people expressing sentiments such as ‘all the money is being earned by outsiders’, and having feelings of exclusion and a lack of fairness. Furthermore, those residents who provide tourism services (such as being a local guide) generally perceive that boutique rural hotels have negatively impacted their livelihoods. This is because, in comparison to regular B&Bs that only provide accommodation, the boutique rural hotels usually provide almost all the services tourists utilize, including food and refreshments, guides, transportation and drivers. This forces local people to work under contract to the boutique rural hotels, which tends to be on less favorable terms than if they would be working for themselves. Some residents, especially those who don’t provide tourism services, stated that they haven’t received any benefits from tourism, and that the accommodation providers are the only ones making money out of tourism, which happens at the expense of local people, especially because tourism has led to local inflation and other social impacts.

Many traditional activities of the local Tujia ethnic group are no longer being practiced (at least not to the same extent) (Li et al. 2023). For instance, tourism has prompted local people to learn and use Mandarin Chinese more extensively, while the Tujia language and local dialects have been neglected, especially by the younger generations. Additionally, certain practices related to natural resources, such as collecting wild plants and hunting, have been prohibited by the Wulingyuan Management Agency. Nevertheless, to increase the potential for ethnic tourism in the village, some accommodation providers have initiated various actions to preserve traditional culture and architecture. For example, one boutique rural hotel invests in the renovation of local traditional dwellings in order to showcase Tujia architecture to tourists. Also, boutique rural hotels may offer experiential activities for tourists seeking to engage in making local traditional handicrafts, such as brocade weaving and pyrography (an art form and craft that etches a design onto wood or other materials by burning). These activities generally enable local artisans to earn income by selling their products. However, this potentially leads to the commodification of artefacts and culture, which also has various negative social impacts (Shepherd 2002; Vanclay 2002; Cole 2007; Richards 2011), but also arguably facilitates the protection and continuation of cultural practices (Li et al. 2023).

Rural tourism has led to improvements in local infrastructure, including new roads, footpaths, open spaces, observation decks, etc. The government has attempted to maintain the integrity of the local architectural style by imposing various restrictions on local dwellings, aiming to create a more picturesque rural landscape. Furthermore, some public services have improved, with the roads being swept and garbage collected regularly. These changes have enhanced the living environment and have been praised by many local people. However, these changes have also attracted much criticism. Firstly, the construction of some public facilities has been on land allocated to local residents, and they have not received fair compensation for the lost use of this land. The requirement for a uniform architectural style has caused discontent amongst some residents because, although the regulation was established after their houses were built or renovated, they were forced to remove anything that did not comply with the prescribed style. Many villagers considered that ‘the government should have planned ahead’ and consulted villagers in advance about the new regulations.

The increasing number of rural accommodation providers and tourists has led to a significant increase in water consumption. As Longweiba relies on spring water, this increased water usage has resulted in water supply difficulties, particularly during summer (the dry season and when there is more visitors). During the fieldwork for this research, due to the drought at the time, at considerable cost and effort, the local government had to transport truckloads of water daily approximately 20 kms to Longweiba. Furthermore, the increase in sewage from the additional numbers of people has resulted in environmental pollution, especially because there is no centralized sewage treatment system in Longweiba. One interviewee stated that ‘the septic tanks already stink, what will it be like in the future, with more people? Sewage treatment will certainly be inadequate if there is no investment in better services’. The sewage issue is a major concern for all local people, with one stating that ‘if this issue is not resolved soon, we will all have to be relocated’. Some rural accommodation providers discharge their toilet and non-toilet wastewater into ponds, onto farmland, or even into creeks upstream from where other households extract water, leading to complaints and conflict. The problem has not yet been resolved, leading to many residents feeling powerless, saying: ‘What can we do? They [the hoteliers] are all influential people, and we can’t afford to provoke them’.

The boutique rural hotels in Longweiba sometimes host television shows or important official meetings. During such occasions, certain categories of residents, such as communist party members, rangers and village committee workers, are usually required by the village committee to assist (beyond their normal duties) in cleaning-up public areas, leading to complaints such as, ‘we perform cleaning services, help the accommodation providers get guests, and make money for them, but we receive nothing in return’.

Most local people referred to tourists as ‘guests’, and all people we interviewed welcomed tourists because of the view that ‘money comes with guests’. The relationship between tourists and local residents was generally good, with only two interviewees mentioning conflicts with tourists, which were due to issues such as tourists trampling on vegetable gardens or inappropriately picking fruit and vegetables without permission. Although local people were aware that rural accommodation can bring some negative social impacts, the majority of local residents had an optimistic attitude about the future of tourism and the tourist accommodation development in Longweiba. They believed that, with the support of government policies, the tourism industry will become more thriving and more sustainable and would provide opportunities for local people to also benefit.

### Social impacts arising from management policies and the governance model

Rural tourism development has triggered significant criticism of local governance and how various government policies have been implemented. The ability to construct and renovate buildings is essential for a viable and vibrant rural accommodation industry, but the strictness of the requirements (especially how it affected people inside the gate at Longweiba) were criticized by many interviewees. A common complaint was the difficulty in obtaining building permits, and the length of time it took to get a permit. One interviewee had to wait for more than a decade to gain permission. Others have applied for permission multiple times, with their requests generally refused. This has created various issues, especially the deteriorating quality of housing, which was a grievance mentioned by many interviewees.

The frequent changes and inconsistency of policies were other issues our interviewees mentioned. Due to the requirements for conservation of the local environment and the possibility that local people might be resettled, prior to 2014, people in Longweiba were not allowed to build new houses or undertake renovation, leading to complaints from many villagers, some of whom had to remain living in old dilapidated houses that did not meet contemporary expectations. However, for people living outside the gate, it was possible to renovate, with some ‘not even having to get an official permit’. Various interviewees mentioned words to the effect that, in 2011 many people living outside the gate were building houses and ‘the local government didn’t control it’. Subsequently, with the Beautiful Countryside and Rural Revitalization policies, construction and renovations of buildings became officially possible, and several buildings were built in 2019. However, this window of permission was short-lived. In 2020, the government again tightened the building regulations, strictly enforcing restrictions on building area and number of floors, with the requirement that the size of houses should not exceed 130 square meters per floor and be no more than two floors high. These specifications did not cater for the needs of farmers, who require considerably more space to store their agricultural equipment. The requirements had the effect of discouraging villagers from renovating due to the belief that, if they renovated their house, this might ultimately result in a smaller living space because they would then have to comply with all the rules including the space restrictions (that they perhaps currently exceeded). Moreover, the constantly changing policies have led local people to question whether their rights and procedural fairness were being respected. One person stated that: ‘some houses have a larger area than permitted, while others are not allowed to exceed the rules even by a small fraction’. Some interviewees expressed the importance of having connections with the officials. There was a perception that ‘without appropriate connections to the village leaders, one cannot get permission to renovate’. They thought this was unfair and improper.

All interviewees living inside the entry gate complained about the housing policies, as it became extremely difficult for them to renovate their house or build anything. An official permit is needed for changes to a house, and all construction materials have to be transported through the gate, which is guarded, so it would be impossible to do such work surreptitiously. Also, the Wulingyuan Management Agency regularly patrols the residential area inside the gate to ensure there is no illegal construction activity.

Before 2020, local people inside the gate were only allowed to have a single-storey house, while those outside the gate could build two-storey houses. A resident living inside the gate said, “this mountainous area is extremely damp, and it is impossible to live in a single-storey house because the walls become completely moldy” making the house uninhabitable. Furthermore, the process of getting permission for renovation or reconstruction of houses was much stricter for residents living inside the gate, with few applications being approved before 2020. The inequality in the rules (between inside and outside the gate) has resulted in severe dissatisfaction among residents, especially those inside the gate, who thought that ‘since the government does not relocate us, we should be allowed to build houses to sustain our livelihoods’. After 2020, it did become possible for residents living inside the gate to renovate their house. However, for various reasons including bureaucratic complexity, only a few people actually did make changes.

Although (at various points in time) the central and local governments have actively promoted rural tourism in Longweiba and throughout China, only the residential areas outside the controlled area have benefited, and the residential areas within the controlled area have not been able to establish tourism facilities. Government investments are primarily directed only towards the development of the residential areas outside the controlled area, resulting in two completely different worlds within the same village: one (outside) characterized as a ‘prosperous world’, and the other (inside) as a ‘traditional village’. The sentiments of the residents inside the controlled area were that ‘we are not allowed to do anything’ and ‘we have been abandoned’. The situation of ‘one village, two policies’ had in fact already existed for 20 years or more, but the recent development of rural accommodation outside the gate has intensified the inequality being experienced. Residents outside the controlled area have more economic opportunities, and some ‘only needed to rent out their houses without doing any work and yet they still earn an annual income’. The perceived inequality has heightened the feeling of ‘lack of fairness’ among residents inside the controlled area, negatively affecting social cohesion in Longweiba, and intensifying conflict between residents inside and outside the gate.

Although tourism in Longweiba has been developing rapidly, an official development plan has not yet been prepared. One villager expressed the view that:

“If, in the past, the government had provided the right direction, such as how to construct rural accommodation, and been clearer, the current situation would be different. Right now, our house is constructed in a certain style, while the neighbor’s house is constructed in another style, simply due to the lack of planning. If the government would have had a plan from the beginning, it would have been better now. Not only for the aesthetics, but all aspects of the village would have been much better.”

Inadequate planning has resulted in the disorderly appearance of the village, which is manifested in the varying architectural styles of the buildings, as well as in inadequate water supply and sewage treatment, which will be an increasing problem with more tourism. The lack of planning has given rise to a sense of uncertainty and insecurity among residents and accommodation providers, who perceive the local government’s stance on tourism to be ambiguous. Therefore, they worry that, at some time in the future, they might have to desist in their tourism activities and/or be forcibly resettled, thus losing any investment they might have made in these activities.

## Discussion and conclusion

As we found in Longweiba in the Wulingyuan World Heritage Site, the development of rural tourist accommodation can have significant positive and negative social impacts on host communities. The notable positive impacts we discovered were on the general economy, heritage, public infrastructure, and the living environment, with the negative impacts being on culture and traditional cultural activities, inequity, intracommunity conflict, environmental pollution, and the lack of inclusion of local people in economic opportunities and overall planning. A key finding was that residents who were not involved in tourism activities experienced more negative impacts than those who were directly connected to tourism. This might be attributable to the extra costs they bear as a result of tourism development, such as an increased cost of living.

The growing number of boutique rural hotels has propelled tourism development in Longweiba, which has increased public recognition of the area, attracted even more visitors, benefitting the boutique rural hotels as well as the small local bed-and-breakfast enterprises. This has contributed to a shift in the focus of tourism in Longweiba away from just promoting the beautiful landscape scenery of the Wulingyuan World Heritage Site towards rural, cultural and ethnic tourism more generally, thus potentially benefiting a wider range of people in the host communities. The increasing number of rural accommodation providers has also stimulated government investment in the region, leading to some improvement in community infrastructure and public facilities, thereby enhancing the local living environment (e.g. clean streets and regular garbage collection), although water and sewage still remain as key issues to be addressed. Although rural tourism has contributed to preserving local culture and heritage in Longweiba, the declining use of the local dialect by younger people highlights the negative consequences of tourism on local culture. Sewage, odor pollution, water supply and water pollution issues have also resulted from the increasing tourism in Longweiba, raising concerns among local residents about their future.

The ongoing viability of tourism remains a key concern for some residents, because they are aware that they receive some benefits from tourism and are thus dependent on it for their continued wellbeing. Some other residents considered that the benefits from tourism are mostly gained by outsiders, and that they are largely excluded from benefiting. We suggest that it would be useful if the local government would provide training in the management and operation of small hotels and B&Bs, and encourage increased entrepreneurialism amongst local people, for example by promoting local food production and establishing local markets.

Having a greater understanding of the negative and positive social impacts from tourism and of how these impacts arise would help in the effective management of tourism at the local level. Even though restrictions on house construction at World Heritage Sites and other protected areas in China and elsewhere are normal (Wang 2021), complaints from residents about these restrictions are longstanding, and the rapid development of tourism has exacerbated these grievances. Similarly, the differing management strategies applied within the village (inside and outside the gate) have intensified community conflicts and dissatisfaction. Residents in areas subject to stricter conservation restrictions experience poorer living conditions, and generally lack opportunities to engage in tourism. They also do not receive government support for infrastructure development. In other words, they bear the costs of conservation and tourism development, yet have limited access to tourism-related benefits, thus widening the gap between those who profit from tourism and those who shoulder its burdens.

The Chinese government is actively promoting tourism to protected areas and rural tourism generally (Ma et al. 2020; Dai et al. 2023). Nevertheless, because of the management arrangements relating to the Wulingyuan World Heritage Site, there are complications and contradictions in Longweiba. Many local people have a strong desire to be a tourist accommodation provider and intend to convert their house into a B&B. When the restrictive housing policy was relaxed in 2014, many residents rapidly acted to provide tourism accommodation and other services. However, there was a long lag time between the announcement of policies supportive of tourism and appropriate changes being made to the local planning laws. This led to much frustration.

There is also an evident contradiction between conservation and community development. While nature conservation is a key focus area for local government, rural revitalization is (or at least should also be) a critical action area for local government (Yang et al. 2021). Creating synergy between economic development and heritage conservation is vital, but difficult to achieve (Heslinga et al. 2020). The local government’s ambiguous management approaches and policies indicate that an appropriate balance is yet to be achieved in the trade-off between nature conservation and tourism development.

Our research findings revealed various shifts in local power dynamics that were induced by the tourism industry. Because attracting external investment is a goal, local governments have supported boutique rural hotels in various ways, with limited consideration being given to other local interests or concerns. This has led to various contradictions, for example the inappropriate discharge of untreated sewage by hotels without intervention by environmental authorities or regulators. Furthermore, nepotism and cronyism are common (Weng and Peng 2014). Another issue was poor information dissemination, and the limited knowledge by villagers of applicable regulations. The rise of tourism has created a new group of local economic elites aligned with local political elites and established developers. Together, they form growth coalitions to promote their mutual interests (Ma et al. 2020). These partnerships have marginalized many local residents, particularly those not directly involved in tourism.

Like much other research, we also found that rural tourism accommodation brings a range of negative and positive impacts to host communities. However, we also found that, for rural tourism, the induced social change processes described by Vanclay (2002) potentially unfold in ways that are different to other forms of tourism (Rosalina et al. 2021). For example, in contrast to large-scale tourism projects (e.g. big hotels), local B&Bs have limited resources and limited local influence. Local rural tourism developments have the potential to promote local participation and local cooperation, especially when initiatives are led by residents and are aligned with local cultural practices. In practice, however, bottom-up tourism developments usually encounter significant obstacles due to fragmented governance structures, unclear regulatory frameworks, and a lack of institutional support.

The ‘one village, two policies’ approach may require rethinking because it creates discrepancies between residents and intensifies community conflict and inequity. To create some balance, we suggest that the local government and management agencies foster ecologically friendly livelihood activities for residents living inside the entry gate, for example, allowing activities like wild honey production, traditional agriculture, artisanal production of Tujia artefacts, or tea production (Su et al. 2019). These traditional activities can promote ethnic tourism and are likely to have other benefits.

Participatory planning is essential to ensure the sustainable development of local tourism. Regulations governing the construction of buildings, maintenance, and the use of buildings are also necessary to prevent over-tourism from compromising Wulingyuan’s outstanding universal values. Community participation and benefit-sharing arrangements must be part of the process (Dixit et al. 2024; Vanclay 2024b). Also, there needs to be consideration about how to manage the effects of local inflation on the host community (Cheer et al. 2019). We believe that some form of community-based management is essential because it promotes community involvement, encourages social equity, empowers local people, and benefits the broader population, and thus builds trust, and likely leads to gaining a social licence to operate for tourism development in the region (Okazaki 2008; Bickford et al. 2017; Burgos and Mertens 2017; Jijelava and Vanclay 2017, 2018; Vanclay 2017; Zhang et al. 2024; Kahangirwe et al. 2025).

Our research was based on a qualitative case study of a single village within the Wulingyuan World Heritage Site. While this allowed for a detailed in-depth understanding of the social impacts at the community level of this village, this village may not be typical of the other villages in or near the Wulingyuan World Heritage Site. Future research could explore the experience of the other villages in Wulingyuan, or other protected areas in China or elsewhere. The specific governance arrangements and cultural practices at play in China are also likely to lead to some differences than what might be found in some other settings. Our study primarily focused on local peoples’ perceptions of the impacts of tourism accommodation development, rather than being an overarching study about tourism generally. For example, our research did not consider other stakeholders, such as tourists, government authorities, or external operators, all of whom would also have views about the effectiveness of tourism arrangements. The non-inclusion of these stakeholder groups limits our ability to undertake a more detailed examination of governance interactions. Future research adopting a multi-stakeholder, multi-level governance perspective would be useful. Also, since there is rapid change, undertaking longitudinal research would also be useful to capture the evolving and dynamic nature of social impacts associated with rural tourism development in protected areas.

## Data Availability

Data will be provided on request.
